# Concerns and Support after One Year of COVID-19 in Austria: A Qualitative Study Using Content Analysis with 1505 Participants

**DOI:** 10.3390/ijerph18158218

**Published:** 2021-08-03

**Authors:** Yvonne Schaffler, Afsaneh Gächter, Rachel Dale, Andrea Jesser, Thomas Probst, Christoph Pieh

**Affiliations:** Department for Psychotherapy and Biopsychosocial Health, Danube University Krems, 3500 Krems, Austria; afsaneh.gaechter@donau-uni.ac.at (A.G.); rachel.dale@donau-uni.ac.at (R.D.); andrea.jesser@donau-uni.ac.at (A.J.); thomas.probst@donau-uni.ac.at (T.P.); christoph.pieh@donau-uni.ac.at (C.P.)

**Keywords:** distress, problem areas, concerns, stress coping, COVID-19 pandemic, Austria

## Abstract

The COVID-19 pandemic and subsequent governmental restrictions have had a major impact on the daily lives of Austrians and negatively affected their mental health. A representative sample of N = 1505 individuals was recruited via Qualtrics^®^ to participate in an online survey between 23 December 2020 and 4 January 2021. A qualitative study design was used to determine the problem areas that emerged since the beginning of the pandemic (question 1), the factors that were the source of the greatest current concern (question 2), the biggest worries when thinking about the future (question 3), and what the most important source of support (question 4) during the pandemic was. The written responses were analyzed using conventional content analysis following a framework for qualitative research and reported in the form of descriptive statistics. Restrictions imposed by the government, sociopolitical developments, work- and health-related issues, and economic disruptions were identified as being the greatest concerns. Conversely, social contacts within and outside the family were the most important source of support, followed by recreational activities and distraction. Greater consideration should be given to psychosocial factors in future decisions to contain the pandemic. More detailed qualitative research, in particular, to collect the personal experience of more vulnerable groups such as young people, women, and the unemployed, is needed.

## 1. Introduction

The infectious disease SARS-CoV-2 not only causes severe illness and an ever-increasing death toll among those infected with the virus but also has a significant impact on public mental health [1,2]. Efforts to mitigate the spread of the disease or to “flatten the curve” have led to unprecedented efforts to institute the practice of social distancing in countries around the world, resulting in lockdowns involving the closure of schools, stores, and cultural facilities as well as travel restrictions [3]. While social distancing prevents an uncontrolled spread of the coronavirus, it is also expected to cause difficulties in many situations and increase feelings of isolation, stress, and frustration [4,5].

In Austria, the government imposed the first nationwide lockdown on 16 March 2020. In early summer, following a decline in the number of people infected, the lockdown was lifted entirely. As the number of people infected with COVID-19 increased again through the summer, a “light” lockdown was imposed in September 2020, followed by a second “hard” lockdown with a curfew and a ban on all events before Christmas, which, with a few brief exceptions, remained in place until 7 February 2021 [6]. In this study, we primarily focused on the second “hard” lockdown after almost one year of the pandemic in Austria.

To date, research into the factors that contribute to either a decline or an improvement in mental health during the pandemic has been predominantly quantitative, focusing on pre-existing hypotheses. Only a few studies have looked more openly into the specific aspects of the multiple fields and sources of stress that the COVID-19 pandemic has the potential to affect, mostly using small samples that support in-depth case-oriented analysis, as did Sun et al., who phenomenologically explored the psychology of 20 nurses caring for COVID-19 patients in China during the early stage of the pandemic. They found that negative emotions were dominant and that positive emotions appeared only marginally. Coping with stress played an important role in maintaining their mental health, such as by writing diaries and letters, breathing relaxation, mindfulness, music meditation, and emotional expression and venting [7]. Another study exploring the experience of COVID-19 during the early stages of the pandemic examined five virtual focus groups with a total of 27 adult participants from the UK. It brought to light the following themes: negative social and psychological impacts of social distancing and isolation; criticisms of government communication around social distancing and isolation; current adherence and non-adherence of self and others; and uncertainty, social reintegration, and the future [8]. Drawing on a sample of 38 individuals from the Israeli public, mostly women, Levkovich and Sinan-Altman found the following sources of participants’ emotional responses and senses of threat: health concerns regarding themselves and their loved ones; employment concerns; problems with children and spouses caused by being together at home; and difficulties involved in working at home [9]. Whitehead et al., on the other hand, whose comparably large sample comprised 825 older adults from the US, identified 20 stress categories and 21 joy/comfort categories from their participants’ written reports in answer to open-ended questions. The most commonly reported stressors were restrictions, concern for others, and isolation/loneliness; the most commonly reported sources of joy or comfort were family/friend relationships, digital social contact, and hobbies.

Whitehead et al. anchored their view on stressors and joys within the transactional theory of stress and coping (TTSC) by Lazarus and Folkman [10], a cornerstone of psychological stress and coping research across multiple fields and disciplines [11]. This theoretical framework, which we also apply to discuss our results, holds that the stress experience depends on the relationship between an individual and their environment. In this view, a potential stressor becomes a negative stressor through two factors: first, when the potential stressor is thought to be relevant for the individual, and second, when the individual thinks that he/she is unable to cope with the potential stressor. For example, the coronavirus itself may be experienced as extremely stressful for someone who is in poor physical health, whereas subsequent lockdown measures and “social distancing” recommendations may be experienced as more stressful to those who lead a life, whether privately or professionally, that is highly dependent on traveling or meeting others. Coping strategies are only used if an event (such as the pandemic) is appraised as stressful [12].

Previous studies that have examined the impact of the pandemic and the government-implemented containment measures on mental health have shown that the pandemic is indeed stressful, including beyond the effects that are directly attributable to the virus such as health and mortality risks [2,13,14]. It is not only “true quarantine” (complete separation and restriction of movement) that has been shown to entail substantial effects on emotional distress and mental health, including depression, generalized anxiety, insomnia, and post-traumatic stress [15]; lighter stay-at-home regulations including personal distancing behavior have also been associated with symptoms of depression, generalized anxiety disorder (GAD), intrusive thoughts, insomnia, and acute stress [16].

In an evaluation of mental health during the first COVID-19 lockdown in Austria, Pieh, Budimir, and Probst [17] concluded that the COVID-19 pandemic and lockdown seem particularly stressful for those whose life circumstances have been affected, such as younger adults (<35 years), women, people without work, and people with low incomes. Furthermore, Pieh et al. found that the reduction in well-being persisted despite the end of the lockdown measures [18] and even six months after the outbreak of COVID-19 [19]. In addition, a longitudinal online survey (with the first wave during the lockdown from 10–30 April 2020 and the second wave after the lockdown from 11–20 June 2020) investigated changes in depression during and after lockdown in Austrian adults. It revealed that more individuals changed from being not depressed during lockdown to being depressed after lockdown, N = 39 (8.8%), as compared with a change from being depressed during lockdown to not being depressed after lockdown N = 21 (4.7%), a phenomenon that, according to a moderation analysis, was due to an interaction effect of stress and loneliness during lockdown [20]. During the second “hard” lockdown surveyed at the end of the first year of the pandemic, Dale et al. [21] observed relatively poor quality of life as compared to both the first lockdown earlier in 2020 and pre-pandemic data. Further analyses indicate that these findings were especially visible in those under age 24, women, single/separated people, those with low incomes, and those who did not partake in any physical activity.

Within the TTSC framework, stress management or coping behavior may be either problem-focused, which means that coping is aimed at changing the source of the stress, or emotion-focused, which means that coping behavior is oriented toward managing the emotions that accompany the perception of stress. For example, a problem-focused strategy would be directed toward taking action to try to eliminate or solve the problem, which is difficult for an individual in a pandemic as the struggle against the pandemic requires an orchestrated effort. In such a situation, coping is expected to be emotion-focused, and known strategies are the seeking of the company of friends or family for comfort and reassurance or oriented toward managing the negative emotions associated with stress [11]. From quantitative studies conducted during the first lockdown in Austria, we know that emotion-focused strategies such as positive thinking, active stress coping, and social support were indeed found to be positive predictors for psychological life quality and well-being and negative predictors for perceived stress, depression, anxiety, and insomnia. Positive thinking was the strongest predictor for psychological well-being, followed by social support, thus indicating that these coping strategies were the most beneficial for mental health [22]. Being in a relationship, however, was not per se associated with better mental health. Instead, it became apparent that the quality of a relationship was essential when it came to the prevalence of depressive symptoms. Compared to no relationship, good relationship quality was a protective factor, whereas poor relationship quality was a risk factor [23]. A UK study into the association of relationship quality during the COVID-19 lockdown also found that living in a good relationship seemed to be an advantage, while individuals with poor relationship quality were particularly affected and drank significantly more alcohol during the COVID-19 lockdown [24]. In a similar vein, Mariani et al., who focused on Italian individuals, showed that depressive symptoms correlated positively with low social support, specifically related to family support [25].

In sum, results from previous studies indicate that the first year of the pandemic was experienced as stressful across nations. Not only physical health and mortality risks but also stay-at-home regulations have shown to be associated with a range of detrimental psychological effects. Sources of stress besides health concerns were concerns clustering around the governmentally imposed restrictions and their direct effects such as loneliness and isolation, as well as work concerns, financial loss, and feeling uncertain about the future. On the other hand, social contacts (digital or otherwise), positive thinking, relaxation, and engaging in hobbies were among the most frequently and successfully utilized means to cope with pandemic-related stress.

### Aims and Questions

In our study, we primarily focused on stresses and resources in a timeframe at the end of the first year of COVID-19 in Austria around Christmas 2020, after the government announced a strict prohibition on larger social events such as family meetings and the celebrations around New Year’s Eve and after the announcement of the existence of a highly contagious mutation of the virus that was expected to spread throughout Europe [26]. A secondary goal was to also look back at problem areas since the beginning of the pandemic in Austria and at worries our participants thought of when they anticipated the future. Like in many other European countries, the first “hard” lockdown in Austria, which lasted several weeks, started in March 2020. All shops (except basic services), cafés, bars, and restaurants as well as the federal gardens had to remain closed. Strict contact and exit restrictions based on the national COVID-19 law came into effect, bringing public and social life largely to a standstill. 

In contrast to previous qualitative studies that drew upon rather small (sub-)samples from the general population, we generated data from a statistically representative sample of the Austrian public. It was our aim to explore the specific burdens of COVID-19, as well as the resources our participants turned to for help in the midst of the second “hot phase” of the pandemic. The following four open-ended questions were designed to shed light on the experience of stressors at the time since the local onset of the pandemic, at the present time, and what stressors participants anticipated in the future, as well as on current sources of support: (Qu.1) Which problem areas have emerged since the beginning of the pandemic? (Qu.2) What currently gives you most cause for concern? (Qu.3) What is your biggest worry when you think about the future? (Qu.4) What is currently providing you with the most support? Since the term “coping” might be associated with mental disorders including depression and addiction, we used the more open-ended expression “support” to encourage participants to think more in terms of everyday life. No word limit was placed on responses. 

As the types of stressors are reported through different time frames (past (Qu.1), present (Qu.2), and future (Qu.3)), a comparison allowed us to observe a subjectively experienced temporal development of concerns. The resulting “texture of sorrows” is strongly informed not only by the psychological state of the Austrian population at the time the survey was open [21] but also by the social distancing guidelines and other policies and messages from the government and the media that were in place at the time of our survey. By exploring the interplay between individuals and their social context, we took into account the fact that a pandemic is inherently a social phenomenon.

## 2. Materials and Methods

### 2.1. Sample

To measure mental health and explore factors that contribute to its deterioration or improvement during the COVID-19 restrictions over the Christmas period 2020 in Austria, a cross-sectional online survey was conducted using Qualtrics^®^ (Qualtrics, 2019). The survey started on 23 December 2020 and ended on 4 January 2021. A Qualtrics panel organized participant recruitment and data collection. The goal was to achieve a representative sample for Austria of at least 1500 participants by age, gender, education, and region, combining age and gender quotas, according to the Eurostat database. Because the survey period was limited, not all the quotas were met; for example, there was a shortage of young males and persons with a high education level (see Supplementary materials, Table S1). The participants of our representative total sample were N = 1505 adults residing in Austria, 49.2% women and 50.8% men, aged 18–24 (10.2%), 25–34 (18.5%), 35–44 (19.2%), 45–54 (21.7%), 55–64 (18.1%), and 65+ (12.4%). The survey reached participants in all Austrian federal states: Burgenland (3.9%), Lower Austria (20.3%), Vienna (23%), Carinthia (6.8%), Styria (14.7%), Upper Austria (15.9%), Salzburg (5.3%), Tyrol (7.6%), and Vorarlberg (3.5%). Levels of education covered were low (2.7%), middle (53.8%), and high (43.6%). A majority of our participants were married or partnered (59%), 29% were single, 9.9% were divorced or separated, and 2.3% were widowed. In terms of income, 8.6% reported earning <EUR 1000 a month, 21.3% earned EUR 1000–2000 a month, 27% earned EUR 2000–3000 a month, 21.3% reported earning EUR 3000–4000 a month, and 21.8% earned >EUR 4000 a month.

### 2.2. Data Collection

While Dale et al. [21] used standardized questionnaires to measure levels of depression, anxiety, sleep quality, well-being, quality of life, and stress, our study was based on the four open-ended questions regarding perceived stressors and aids: (Qu.1) Which problem areas have emerged since the beginning of the pandemic? (Qu.2) What currently gives you most cause for concern? (Qu.3) What is your biggest worry when you think about the future? (Qu.4) What is currently providing you with the most support? By giving our study participants the opportunity to spontaneously answer what came to their mind first, without having been presented with a pre-determined set of answers, we were able to examine their individual experiences in their own context [27], thus achieving more depth as to why this population has been adversely affected and what has been of greatest help in this difficult situation. Our participants’ written responses ranged from single-word answers to full paragraphs. Both the original questions and answers were presented in German. When answering our questions, the participants were forced to enter at least one letter into each free text field; otherwise, they could not move on to the next question. Some of them chose not to answer a question by typing answers such as “x” or “no answer”.

### 2.3. Analysis

We applied a conventional approach to content analysis [28] with subsequent quantification of qualitative categories [27]. Two authors (A.G. and Y.S.) first read all the data to achieve immersion and obtain a sense of the whole, then each answer was read word by word to derive inductive codes by paraphrasing quotations to characterize their content. We used the software ATLAS.ti, assigning each response appearing in the data to at least one code, thus developing a list of codes (or subcategories). In a second step, the larger numbers of codes/subcategories were subsumed under a smaller number of more abstract categories (or themes). This second step was undertaken by each coder separately for all four questions, and then the separate lists of categories/themes were iteratively compared, discussed, adjusted, and organized into a final structure of categories/themes and subcategories, which we refined until the goals of the study were achieved. As participants were allowed to report more than one answer, multiple codes assigned to each response were allowed. We did not limit the number of permitted answers or codes (although there were never more than four answers, and thus codes, per participant, see below). In the next step, the strength of the categories/themes was defined depending on the frequency with which the underlying subcategories were mentioned so that the magnitude of the individual phenomena appears more clearly.

Since the style of our survey often yielded buzzwords rather than entire sentences, and since our participants’ statements greatly varied in terms of their accuracy, our thematic clustering turned out broad, particularly in three areas: “restrictions”, “sociopolitical developments”, and “physical health”. In the first case, we grouped both imprecisely reported single words or expressions, such as “restrictions” or “COVID-19 measures”, and more precisely reported expressions, such as “loneliness” or “wearing of masks” within one category. The precondition for classification in this category was that the expressions directly referred to restrictions imposed by government actions to contain COVID-19. In the second case, our clustering style was influenced by the fact that our participants’ statements, more often than not, were difficult to assign to separate categories. For example, concerns regarding the labor market often appeared connected to statements regarding the general economic situation or to the assumption of political mismanagement. The same was true for the third case: we clustered the otherwise separate categories “concerns for one’s own health” and “concerns for the health of others” within only one broad category named “physical health” because a portion of the answers only mentioned “infection with COVID-19”, which could refer to one’s own infection or to infection of others.

### 2.4. Ethics

This study was conducted in accordance with the Declaration of Helsinki and approved by the Ethics Committee of Danube University Krems, Austria (ethical number: EK GZ 26/2018–2021). All participants gave electronic informed consent to participation and to completing the questionnaires. Data were collected anonymously without IP addresses or GPS tracking, and this procedure was approved by the data protection officer of Danube University Krems.

## 3. Results

In the following, we report results to questions 1–3, which refer to (Qu.1) “problem areas that have emerged since the beginning of the pandemic”, to (Qu.2) “the greatest current concern”, and to (Qu.3) “the greatest anticipated future worry”. Whereas Qu.1 and Qu.2 yielded answers that referred to past and current experiential aspects of the pandemic, Qu.3 brought to light expressions of further thoughts about things that could happen. 

**Figure 1:**
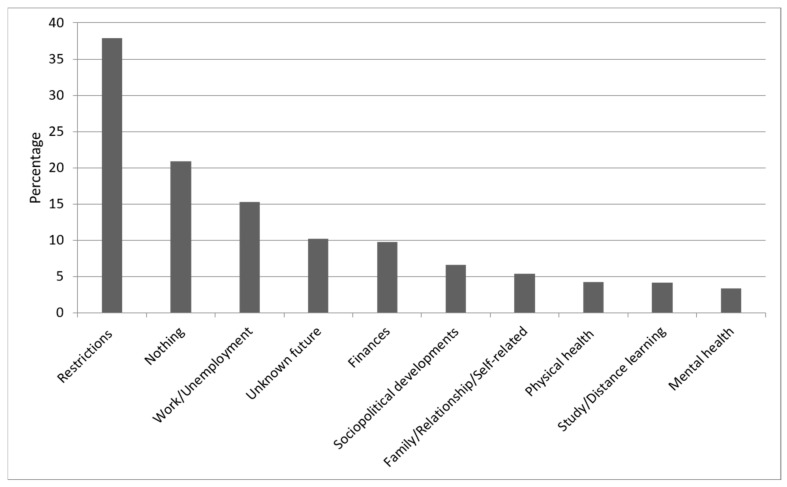
Sources of problems since the beginning of the pandemic. The percentages of participants reporting each main category of problem area that emerged from the data for the first question: “Which problem areas have emerged since the beginning of the pandemic?”.

**Figure 2:**
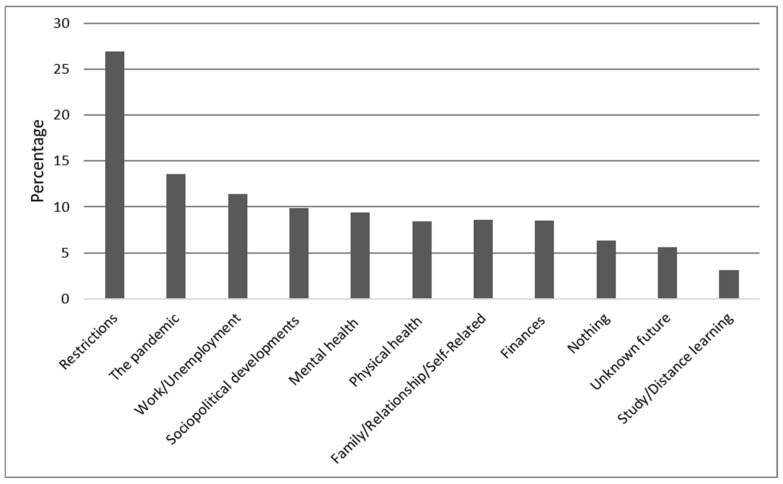
Sources of current concerns. The percentages of participants reporting each main category of concerns that emerged from the data for the second question: “What is currently causing you the greatest concern?”.

**Figure 3:**
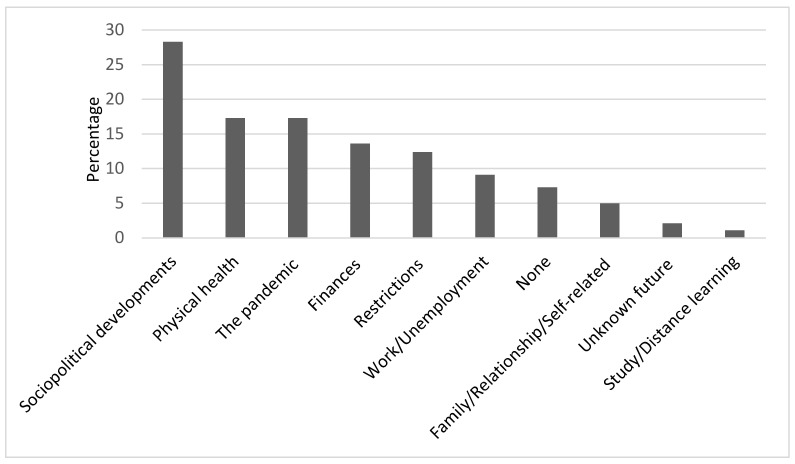
Sources of greatest concern for the future. The percentages of participants reporting each main category of concern that emerged from the data for the third question: “What is your biggest worry when you think about the future?”.

**Figure 4:**
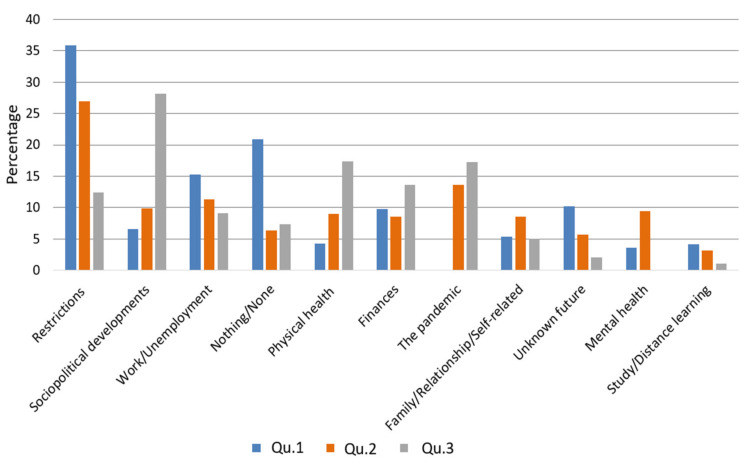
Percentages of main categories for questions 1–3. The percentages of participants for each question (1–3) reporting each main category of response, ordered by total. Qu.1 refers to “problem areas since beginning of pandemic”; Qu.2 to “greatest current concern”; Qu.3 to “greatest future concern”.

Although the three questions have different emphasis regarding their reference to a certain time frame (past (Qu.1), present (Qu.2), and future (Qu.3); see Figure 1, Figure 2 and Figure 3), our thematic clustering yielded similar categories/themes in response to all three questions. We therefore also visualized the data in response to questions 1–3 within one comparative bar chart to show how they are ordered according to their total sum, how the magnitude of themes changes across time frames, and to also report on shifts in content across time frames (see Figure 4). 

**Figure 5:**
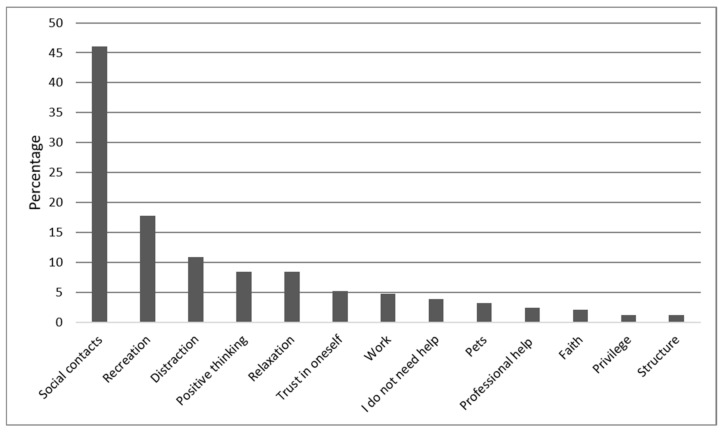
Sources of greatest current support. The percentages of participants reporting each main category of response that emerged from the data for the fourth question: “What is currently providing you with the most support?”.

Subsequently, we proceed to report on answers to question 4 referring to “sources of greatest current support” according to the percentage of participants reporting each category/theme (Qu.4; see Figure 5).

### 3.1. Results for Question 1: Which Problem Areas Have Emerged since the Beginning of the Pandemic?

#### 3.1.1. Study Sample for Question 1 (N = 1456)

Of N = 1505, N = 49 (3.2%) did not reply to question 1. Of the remaining N = 1456, N = 304 (20.9%) reported that they had not experienced any problem areas since the beginning of the pandemic. N = 1376 (94.5%) participants gave only one answer, although multiple answers were possible. A maximum of four codes was assigned to each response to question 1. The results for question 1 are summarized in Figure 1 and are now described in more detail.

#### 3.1.2. Restrictions (37.9%)

The most frequently mentioned problem areas that have occurred since the beginning of the pandemic concern the consequences of government restrictions aimed at containing the spread of the coronavirus. N = 552 (37.9%) reported problems such as “visiting restrictions for children and grandchildren” or social isolation due to the lockdown. In addition, being banned from visiting hospitals and nursing homes and not being able to plan a vacation or take trouble-free trips, as well as “breathing problems when climbing stairs and glasses constantly fogging due to the mandatory wearing of a mask”, were reported as problems.

#### 3.1.3. Work/Unemployment (15.3%)

Other areas reported as problems by N = 223 (15.3%) participants concerned work and unemployment, such as not being able to change jobs for fear of the future and being tied to an employment contract for several years. Fear of losing a job or insecure employment, the difficult situation for career entrants in finding a job or a temporary job, and the lack of jobs in the hospitality industry were also all mentioned. Likewise, restructuring of jobs/industries so as to save businesses and, in the case of unemployment, the difficulty in finding a job were also reported.

#### 3.1.4. Unknown Future (10.2%)

Another problem area concerns the unknown future, as was endorsed by N = 149 (10.2%) participants, who reported uncertainty and fear of the future, boredom, a loss of concentration and motivation, and a feeling of helplessness because there was nothing that they could do about the pandemic and its consequences.

#### 3.1.5. Other Problems

Further categories refer to financial problems and money worries associated with the pandemic as mentioned by N = 142 (9.8%). Among those concerns were financial losses due to low income and worries about the future of the participant’s business. N = 96 (6.6%) associated such problem areas related to government policy decisions regarding the pandemic. Mention was made, for example, of “the disproportionate and incomprehensible measures taken by the government” or a “loss of confidence” in politics. N = 78 (5.4%) identified family, relationship, and self-related problems such as “care of family members” or the different ways of thinking about the current situation and the measures adopted by the government, which led to disputes in relationships or within families. Concerns about their own physical health or that of their relatives during the pandemic were reported by N = 62 (4.3%), who expressed comments such as “my daughter’s family has corona and I am afraid I could be infected too” or “worry about the health of the parents”. Changed working or schooling conditions, such as home schooling, distance learning, and working from home were found to pose a challenge to N = 61 (4.2%). Pandemic-related mental health problems were reported by N = 53 (3.4%), such as “depression” or “insomnia”.

### 3.2. Results for Question 2: What Is Currently Causing You the Greatest Concern?

#### 3.2.1. Study Sample for Question 2 (N = 1471)

Of N = 1505, N = 34 (2.2%) did not reply to question 2. Of the remaining N = 1471, N = 93 (6.3%) reported that nothing was causing them concern. N = 1378 (93.7%) participants gave only one answer, although multiple answers were possible. A maximum of four codes was assigned to each response to question 2. The results for question 2 are summarized in Figure 2 and are now described in more detail.

#### 3.2.2. Restrictions (26.9%)

N = 396 (26.9%) reported being concerned as a consequence of the mandated restrictions and resulting confinement. This category covers a lack of social and community contact that occurred due to the introduction of the second lockdown: not being able to meet family or friends, not being able to go to concerts, restaurants, or the theatre, not being able to travel, and the feeling of a loss of freedom. Moreover, participants also reported being affected by loneliness and by having to wear a mouth-and-nose mask in public.

#### 3.2.3. The Pandemic (13.6%)

The second-largest category in the responses to our question, reported by N = 200 (13.6%), was the emergence of the pandemic. This category is composed of single answers that consisted exclusively of one word, for example, “Corona”, “COVID”, “Pandemic”, “Virus”, or “Vaccination”.

#### 3.2.4. Work/Unemployment (11.4%)

The third-largest category, named by N = 167 (11.4%), concerned the work situation and unemployment related to the pandemic and restrictions. The most frequently reported changes in the work situation were the occurrence of stress due to either overtime at work or reduced hours or furlough (so-called “Kurzarbeit”), increased aggression at work, and worries about losing the job, as well as unemployment due to measures adopted or long-term unemployment and the difficulties in finding a job in times of a pandemic.

#### 3.2.5. Other Concerns

A further category relates to concerns associated with current sociopolitical developments. N = 146 (9.9%) reported that government decisions caused them concern because they could lead to restrictions on civil liberties. They also stated that the measures would lead to economic and social changes that were worrisome. Pandemic-related mental health concerns were reported by N = 139 (9.4%), such as “I feel anxious that my depression will return” and a feeling of “restlessness” or “insomnia”. Physical health stresses were mentioned by N = 133 (9.4%) such as “fear of contracting the virus and thus endangering my own health or worrying about the health of others” and “fear of infecting my own parents or elderly clients”. Emerging family, relationship, and self-related problems were mentioned by N = 126 (8.6%), such as “unexpected separation” or the sudden split in the family due to a disagreement about how to handle the pandemic or “little control in life due to babies and toddlers” and “a lack of childcare”. N = 125 (8.5%) were concerned about the financial burdens or the economic impact of the pandemic such as “I won’t have enough money when I retire” or money worries that arose either in the wake of unemployment or due to low income. Uncertainty about the unknown future related to the pandemic and its effects was reported by N = 83 (5.6%), such as “uncertainty about how long the pandemic will last” or “not knowing what will happen with COVID-19 and how long antibodies will work if someone falls ill with Corona”. For N = 46 (3.1%), the impact of COVID-19 measures on studying, distance learning, school, and home schooling was very stressful, with such replies as “not being able to live as I did before, exam stress at university as well as the pressure I put on myself, home office and doing everything from home”.

### 3.3. Results for Question 3: What Is Your Biggest Worry When You Think about the Future?

#### 3.3.1. Study Sample for Question 3 (N = 1424)

Of N = 1505, N = 81 (5.4%) did not reply to question 3. Of the remaining N = 1424, N = 104 (7.3%) reported that they had no worries. N = 1095 (76.9%) participants gave only one answer, although multiple answers were possible. A maximum of four codes were assigned to each response to question 3. The results for question 3 are summarized in Figure 3 and are now described in more detail.

#### 3.3.2. Sociopolitical Developments (36.5%)

N = 401 (28.3%) were worried about current sociopolitical developments. Within this category, we subsumed a variety of concrete worries concerning economic shifts, such as demonetization, the repayment of government debt, and the future of the labor market, which formed the largest subcategory, with N = 155 (10,9%). Additionally, we included an array of less specifically expressed fears regarding the weakening of government facilities, in particular, the health care or education systems, which N = 85 (6%) also expressed as only a vague concern about “the future of our children” or “the future of coming generations”. Worries were also pointed out by N = 73 (5.1%) regarding future responsibility, e.g., that people “will not learn from the pandemic”, that “humanity will destroy itself”, or referred to a fear of social unrest as a consequence of the pandemic. A loss of trust in government policy was found to be another, yet smaller, subcategory. Further worries referred to sociopolitical developments that were not related to the pandemic, such as environmental pollution or climate change, and fear of uncontrolled immigration.

#### 3.3.3. Physical Health (17.3%)

The second-largest theme regards worries for the participants’ own health, with N = 183 (12.9%) stating that they were generally worried about remaining healthy or that they were concerned about other, non-pandemic-related health problems, of which they feared that these ailments would not be treated properly as a consequence of a too-strong focus of medical personnel on the pandemic. Questions were asked, such as “when will my kidney transplant finally happen?” Within the same category fall also worries about the health of others, in most cases family members such as one’s parents or partner N = 64 (4.5%). More unspecific worry about “infection with COVID-19” was mentioned by N = 17 (1.2%).

#### 3.3.4. The Pandemic (17.3%)

A similarly sized category of worry for the future relates to the pandemic itself and to how it will progress. Major worries that we identified from our data concerned doubts that there would ever be a return to normality or that vaccination or other types of measures could really end the pandemic. Other pandemic-related worries that we observed referred to virus mutations potentially leading to a “pandemic within the pandemic”, to one wave of COVID-19 following another, as the current pandemic would not come to an end, or that other pandemics with different viruses might follow the present one.

#### 3.3.5. Other Worries about the Future

These three main themes were followed by worries regarding people’s personal financial situation, as was mentioned by N = 194 (13.6%), who reported being worried about an ongoing loss of income, the fear of losing their homes due to an inability to repay their debts, and a worry that the financial situation would make it difficult to earn enough to ensure a good pension later. Concerns about restrictions in the future were expressed by N = 176 (12.4%), some of whom feared long-term political consequences, such as that the measures to contain the pandemic will change the legal situation after the pandemic and cause a loss of liberty and rights. Other worries within this category refer to ongoing isolation and loneliness caused by ongoing restrictions such as social distancing, including the fear of dying alone. Further restriction-related worries refer to the introduction of a mandatory vaccination or an ongoing travel ban. Work-related worries were mentioned by N = 129 (9.1%), including a fear of losing their job or that they would be further disadvantaged by a change in working conditions. N = 71 (5.0%) expressed worries about a variety of family-relationship- or self-related issues ranging from a fear of conflicts within their families and among friends to being overwhelmed by the workload they had to cope with. N = 30 (2.1%) reported worrying about not being able to make plans for the future, and N = 15 (1.1%) mentioned worries about not being able to finish their education or training due to the current conditions within the educational system.

### 3.4. Magnitude of Phenomena in Responses to Questions 1–3

Across time frames, the “restrictions” theme scored highest in response to Qu.1 (37.9%), which aimed at exploring problem areas since the beginning of the pandemic. “Restrictions” also scored high in response to Qu.2 (26.9%) about the greatest current burden, but only 12.4% mentioned “restrictions” as their greatest concern about the future, as we asked for in Qu.3. When looking at how the content of the “restriction” category shifted over time frames, we found that responses to Qu.1 and Qu.2 centered more on visiting bans, social isolation, and the wearing of masks, whereas responses to Qu.3, which was directed toward the future, tended to cluster around the concern that the containment measures could remain in place for a long time, thus requiring a shift in the legal situation and possible detrimental political consequences or around the issue of mandatory vaccination.

The overall second strongest category was that of “sociopolitical developments”, which scored highest in response to Qu.3 (28.3%) about our participants’ greatest future concerns, whereas only 6.6% mentioned it in response to Qu.1 and only 9.9% in response to Qu.2. Regarding content, the category includes dissatisfaction with political decisions and loss of trust in politics, as well as the fear of detrimental effects on the social and economic system. Responses to Qu.3 shift in content toward concerns about what social, economic, and educational conditions today’s youth and subsequent generations will find.

The overall third strongest category concerned the theme of “work/unemployment”, which appears in decreasing proportions from 15.3% (Qu.1) to 11.4% (Qu.2) to 9.3% (Qu.3). Although “work/unemployment” does not change greatly in magnitude across time frames, it appears to be a pressing issue more often in reference to problem areas since the beginning of the pandemic than as a current or future concern. Content-wise the category shifts from more fears of losing or not finding a job (Qu.1) to more experience of stress due to stressful working conditions (Qu.2). Regarding future concerns (Qu.3), the main subtheme was, again, losing one’s job.

The category “nothing/none”, which refers to no past problem areas, no current concerns, and no future concerns, is strongest in response to Qu.1 (20.9%), whereas the category shows much weaker magnitudes in response to Qu.2 (6.3%) and to Qu.3 (7.3%).

“Physical health” stresses are more pronounced in reference to Qu.2 (9.4%) and Qu.3 (17.3%) than to Qu.1 (4.3%). Across time frames, worries about one’s own health and the health of others were expressed. An additional subtopic that appears in response to Qu.3 regards falling ill with or the treatment of other ailments than COVID-19 and worries that they might not be treated properly as COVID-19 continues to be a major focus.

“Finances” ranged similarly from 9.8% (Qu.1) to 8.5% (Qu.2) to 13.6% (Qu.3). Although the main topic across all time frames was “not having enough money for maintaining one’s lifestyle or business”, there was a thematical shift toward more distant future worries about receiving a pension, living in poverty in old age, or being able to continue paying off the house.

The “pandemic” category could not be found in response to Qu.1 about past problem areas but was rather strong in response to Qu.2 (13.6%) and to Qu.3 (17.3%). In terms of content, the “pandemic” category shifted from mere buzzwords such as “Corona” or “Pandemic” in response to Qu.2 regarding the greatest current concern to more concrete future pandemic-related worries in response to Qu.3, such as regarding virus mutations, future waves of the pandemic, or doubts that the pandemic can be ended with the measures available.

The category clustering around “family/relationship/self-related” issues, ranged from 5.4% (Qu.1) to 8.6% (Qu.2) to 5.0% (Qu.3), showing its greatest magnitude at the current point in time. Whereas interpersonal conflicts and feeling overwhelmed were mentioned in response to all three questions, the issue of overstrain due to lack of childcare was most prominently expressed in response to Qu.2.

The category “mental health” was stronger in response to Qu.2 (9.4%) about what is causing the greatest current concern than in response to Qu.1 (3.4%) about problem areas since the beginning of the pandemic. It was not found to be a category in response to Qu.3. Content-wise it revolved in both cases predominantly around depression and insomnia.

The category “unknown future” showed the greatest magnitude in response to Qu.1 (10.2%) and appeared in decreasing proportions in response to Qu.2 (5.6%) and Qu.3 (1.1%). In terms of content, responses to all three questions were different. Whereas responses to Qu.1 refer to feelings of boredom, uncertainty, or helplessness in the light of great perplexity, as people did not know what to expect from the future, responses to Qu.2 asking for the greatest current concern dealt in a more concrete way with future prospects such as the duration of the pandemic and immunity to COVID-19. In response to Qu.3, answers subsumed under “unknown future” referred to the inability to make plans in the light of the ongoing uncertainty.

“Study/distance learning” was endorsed with decreasing magnitude by 4.2% (Qu.1) to 3.1% (Qu.2) to 1.1% (Qu.3).

### 3.5. Results for Question 4: What Is Currently Providing You with the Most Support?

#### 3.5.1. Study Sample for Question 4 (N = 1449)

N = 56 (3.7%) out of N = 1505 participants did not answer the fourth question. Of the remaining N = 1449, N = 77 (5.3%) reported that nothing was providing them with support. N = 56 (3.9%) stated that they did not need support. N = 1275 (89.5%) participants gave only one answer, although multiple answers were possible. A maximum of four codes was assigned to each response to question 4. The results for question 4 are summarized in Figure 5 and are now described in more detail.

#### 3.5.2. Social Contacts (46%)

When asked what was currently providing most support, N = 667 (46%) referred to social contacts, either explicitly mentioning their partner, N = 186 (12.9%), or referring more generally to their “family” or to family members in particular such as their children, siblings, parents, or grandparents, N = 290 (20%). When friends or colleagues were cited as a main social resource during the pandemic, N = 191 (13.2%), our study participants often added that they were engaging with them over the phone or the internet or while going for a walk.

#### 3.5.3. Recreation (17.8%)

N = 258 (17.8%) cited recreational activities as the greatest source of support, referring primarily to outdoor exercise such as walking, hiking, jogging, or cycling or indoor exercise including yoga and aerobics, N = 158 (10.9%). Other recreational activities mentioned by our participants were hobbies such as reading, listening to music, making music, needlework, or gardening, N = 101 (7%).

#### 3.5.4. Distraction (10.9%)

N = 168 (11.6%) found distraction from the pandemic to be of greatest support, such as watching television, engaging in video games, internet surfing, or sleeping a lot. We defined drinking alcoholic beverages or smoking cigarettes, as was endorsed by N = 22 (1.5%), as a subcategory of distraction from the pandemic.

#### 3.5.5. Other Sources of Support

Positive thinking was endorsed by N = 122 (8.4%) stating for example, “I hope for a better future”, “I believe in myself”, or “I am an optimist”. In addition, conscious relaxation through meditation, drinking a cup of tea, taking a hot bath, or breathing exercises was mentioned by N = 121 (8.4%), while “trust in oneself” helped N = 76 (5.2%), which they expressed by referring to their “own strength”, “primal trust”, knowledge, or “fighting spirit”. “Work” or “working more” was cited as most helpful by N = 68 (4.7%). N = 46 (3.2%) mentioned pets such as cats, dogs, or horses as their greatest source of support during the pandemic. N = 35 (2.4%) mentioned professional help from their doctor, psychologist, or psychotherapist. “Trust in God” and other affirmations of religious faith were reported by N = 31 (2.1%). N = 18 (1.2%) found it helpful that they enjoyed a privileged life situation including a beautiful home or a regular income, and N = 17 (1.2%) engaged in actively structuring their time or living environment, which they said involved making sure that they made a plan for the day or that they engaged in domestic work or decorating their home.

## 4. Discussion

Our findings primarily reflect a snapshot of the perceived impact of COVID-19 during social distancing in a representative Austrian sample of 1505 participants. To provide a more contextual exploration of stressors, we also looked into the development of concerns from the beginning of the pandemic to future concerns (past (Qu.1), present, (Qu.2), and future (Qu.3)), as subjectively experienced by our participants. The resulting picture was strongly informed by our participants’ situation during the time of the survey as a second “hard” lockdown took place around Christmas 2020, media coverage that circled around social distancing policies, newly detected dangerous virus mutations and death rates, and our participants’ deteriorating mental health.

Overall, our participant responses showed that the main sources of distress or challenges clustered around the pandemic-induced restrictions and the resulting confinement, including a lack of social contact, both in response to the question that asked to look back to the beginning of the pandemic and identify problem areas that had since emerged (Qu.1) and about the most significant current concern (Qu.2). Worries about the future (Qu.3) tended to cluster primarily around expected detrimental effects on sociopolitical developments, that is, the development of the economy, the labor market, democracy, and governmental institutions. Regarding what our participants saw as providing the most support (Qu.4), the sources reported tended to be social relationships mostly within, but also outside, their families.

### 4.1. Stressors (Questions 1–3)

As we expected in the light of the TTSC framework [10] and previous findings that indicate that the coronavirus pandemic is a severe global crisis that affects both physical and psychological health, we found that all reported sources of distress at the end of the first year of the pandemic, except two minor subthemes of the “sociopolitical developments” category, which refer to the climate crisis and to the issue of immigration, were related to COVID-19.

In the following, we discuss our participants’ concerns according to the overall strength of categories/themes across time frames (past (Qu.1), present (Qu.2), and future (Qu.3)) as shown in Figure 4; what we think are possible explanations for their proportions and shifts in content; and how the themes we found relate to previous findings.

According to the frequency of our participants’ responses, the primary sources of distress or challenges clustered around the “restrictions” imposed by the government, which led to a lack of social contact, to loneliness (as a consequence of a lack of social contact), or to restrictions on the individual’s cultural life. Given that “quarantine means a loss of control and a sense of being trapped”, which is heightened if families have become separated [29], it does not come as a surprise that “restrictions” had the highest overall score of all stress themes. It not only shows that “restrictions” were the most stressful theme for around one-third of our participants, both since the beginning of the pandemic and currently (that is, around Christmas 2020), but that 12.4% proceeded to report them as their greatest future worry. It is important to note that the future worry category has shifted content-wise from an emphasis on suffering from social isolation (Qu.1 and Qu.2) to seeing the measures to prevent the pandemic as a potential instrument for future political oppression (Qu.3).

In a similar vein, “sociopolitical developments”, which was the overall second strongest category, had a surprisingly high amplitude in response to Qu.3 about our participants’ greatest future worries. It shows that about one-third of our participants feared that the pandemic and its management will have substantial negative effects on the financial and labor market and that negative sociopolitical consequences might ripple into the lives of future generations. It is surprising that rather abstract worries about unknown future scenarios outweigh more concrete sorrows concerning people’s personal work or financial situation. It may be the case that responses within both the “restrictions” as well as the “sociopolitical developments” categories reflect not only fear and worry but also complaints toward the government. The underlying feeling might be anger that distracts from deeper fears. Angry reactions may also be linked to frustration over prolonged public health measures and indicative of people’s struggles to comply. Moreover, since Austria was experiencing a second, much stronger wave of the pandemic at the time of our survey, it is likely that a portion of our participants thought that the government was not able to control the pandemic despite the repeated imposition of harsh social-distancing measures. An alternative or additional explanation could be that individuals in general are more focused on potential negative future events since the pandemic, as suggested by Niziurski and Schaper. They found that their participants thought more negatively of their futures than their pasts (as in opposition to what was found prior to the pandemic), which the authors think is related to their participants’ current high level of psychological distress [30].

The reported stressors regarding the third overall largest category “work/unemployment” reflect the socio-economic implications of the coronavirus pandemic, as pointed out by Nicola et al. [31], who stated that workers around the world have been placed on temporary or permanent leave and that several economic sectors are in a state of crisis. The fact that “work/unemployment” appears to be a pressing issue more often in reference to problem areas since the beginning of the pandemic (15.3%) than as a current (11.4%) or future (9.3%) concern might have to do with successfully implemented federal programs to cushion the devastating effects of the pandemic on the labor market in Austria. However, even if many people did not lose their job as they were sent to reduced hours or to working from home, responses to Qu.2 clearly show that people were burdened by the changed and often more stressful working conditions, as working from home was common in most sectors and since schools remained closed. Another possible or additional explanation for “work/unemployment” appearing as a more important category in the past than currently and looking forward could be that around Christmas other issues appeared more pressing, such as new virus mutations that had spread to Austria [26], which would also explain the appearance of the rather strong category “the pandemic” in response to Qu.2 (13.6%) and to Qu. 3 (17.3%) but not in response to Qu.1.

It is striking that the category “nothing/none”, which refers to no past problem areas, no current concerns, and no future concerns, is strongest in response to Qu.1 (20.9%), whereas the category shows much weaker magnitudes in response to Qu.2 (6.3%) and to Qu.3 (7.3%). Although we are cautious about drawing conclusions about a temporal development, we think that this might, at least partly, be an indication that the experienced pressures have increased over time, which is supported by Dale et al. [21], who conducted quantitative analyses with the same sample as we did. They found that 25.1% were over the threshold for moderate depression and that mental health declined compared to both the first lockdown earlier in 2020 and pre-pandemic data.

“Physical health” stresses were more pronounced in reference to Qu.2 (9.4%) and Qu.3 (17.3%) than to Qu.1 (4.3%), which could be indicative of increased worry over health issues, as the number of cases continued to rise around the world at the time of our survey. It could also have to do with the ongoing emphasis on the pandemic within the health system, such that after almost one year of living in the pandemic and worrisome news from around the world regarding new virus mutations, more individuals have started to worry about their future medical care. We assume that particularly those who suffer from chronic disease were affected by future worries about their physical health.

People’s personal “finances” as a theme, ranging from 9.8% (Qu.1) to 8.5% (Qu.2) to 13.6% (Qu.3), probably most concerned those who had to cut back on their working hours or those with an income that was already low before the pandemic. The fact that future worries about mortgages or the prospect of old-age poverty were added thematically in response to Qu.3 might have to do with the perception that at the time of our survey, the pandemic would likely not come to an end soon, which might have led to the assumption that jobs may be lost forever and that austerity packages are on the government’s agenda. Since not everyone is able to face or express one’s fears head-on, we think that a number of personal existential worries are in fact hidden within statements about the “restrictions” or consequent negative “sociopolitical developments” and thus are subsumed under these categories.

The “pandemic” category, as already mentioned, does not appear in our interpretation of answers to Qu.1 about past problem areas, which, we think, is because answers referring to the beginning of the pandemic rather reflected great perplexity and associated feelings, as it was still unclear whether or not the pandemic would last for long and how serious it was. We thus subsumed them under the category “unknown future”, which shows the greatest magnitude in response to Qu.1 (10.2%), as compared to the other questions. Throughout the pandemic, however, the seriousness of the situation became increasingly evident, which 13.6% of our participants expressed by stating that “Corona” was their greatest current concern. Around 17% expressed their future “pandemic”-related worries clearly in light of the media news about viral mutations and further and stronger waves of the pandemic in different countries at the time of our survey. Since studies also show that parallel with the escalation of the pandemic, fear and worries regarding COVID-19 increased significantly over time [32,33], it is likely that the growing concern in this category might not only be a subjectively experienced but a truly existing trend.

The category clustering around “family/relationship/self-related” issues, ranging from 5.4% (Qu.1) to 8.6% (Qu.2) to 5.0% (Qu.3), shows its greatest magnitude at the current point in time. This development might indeed be due to increased interpersonal stresses and an increased mental health burden from Qu.1 to Qu.2 (again, see Dale et al. [21]). It is also worth noting that at the beginning of the pandemic, particularly depressive and anxious patients at times experienced the lockdown as a relief since it calmed down their hectic and stressful everyday lives [34]. An alternative explanation for the weakness of the “mental health” category in response to Qu.1 or its inexistence in reference to Qu.3 could be that one’s emotional well-being, since it is not a concrete event but rather a feeling at the “fringe of consciousness” (and thus pre-reflective) [35], does not come to mind first when thinking about the past or future.

We think that the category “study/distance learning” in reference to worries about the future (Qu.3) might be weak (1.1%) either because other worries appeared more pressing at the time of our survey, such as the development of the pandemic and associated health worries, or because the theme may be partly hidden within the more general category of concerns about future “sociopolitical developments”, of which a number of statements referred to the future of the educational system.

In sum, our findings indicate that the mentioned stressors almost exclusively refer to the pandemic and its consequences, in particular to “restrictions” and “sociopolitical developments”. They support findings from other authors and countries, according to which quarantine measures and consequent social distancing, work/unemployment concerns, financial losses, health concerns, interpersonal conflict, and uncertainty about the future are among the most frequently mentioned stressors [8,9,15].

We are aware of the fact that a comparison of magnitudes of categories/themes across time frames does not allow us to draw valid conclusions about a true temporal development of concerns from the beginning of the pandemic to what lies ahead. What we could do was trace our participants’ subjective assessment of how their concerns developed, although what they observed was sometimes consistent with observed trends in the literature.

### 4.2. Sources of Support (Question 4)

As was to be expected from a pandemic, which is a problem that cannot be resolved individually, the sources of support that emerged from our data tended to fit within the category of emotion-focused coping, that is, behaviors that aim at adjusting one’s emotions or perspective of the stressor. The spectrum of emotion-focused strategies is quite broad, including strategies such as seeking social support, denial, focusing on and venting emotions, and positively reinterpreting events. Within the framework of our study, we identified the following categories/themes, which we classify as emotion-focused strategies: social support through family, partner, or friends and colleagues (46%), recreational activities such as indoor or outdoor sports or hobbies (17.9%), a positive attitude (13%), distraction (11.6%), work (4.7%), pets (3.2%), seeking professional help (2.4%), seeking support in faith (2.1%), being thankful about one’s privileged lifestyle (1.2%), or structuring one’s day (1.2%). These coping actions used help our participants adjust their own emotions rather than fixing the problem itself, although we would like to note that we cannot be sure that indeed every type of behavior, which we subsumed under the categories “social support” or “work”, is only emotion-focused and not (also) problem-focused, as some individuals might (also) have discussed or worked on how to successfully manage the pandemic.

We furthermore classify almost all coping strategies we found under the umbrella of emotional approach coping, which refers to a subset of emotion-focused strategies. Emotional approach coping tackles the underlying emotional problem in an active, dynamic fashion [36] and contrasts with avoidant strategies that cope in a disengaged way, as they avoid the problem and/or its emotional consequences [37]. In fact, the only category we found that clearly falls within avoidance coping is that of “distraction” (10.9%), which includes sub-categories such as media consumption, sleeping a lot, or substance use.

Our findings align with the TTSC expectation that, for stressors outside an individual’s personal control, such as a pandemic, emotion-focused coping behaviors will be utilized more and will yield a better result than problem-focused strategies [10]. Moreover, when a problem cannot be fixed or changed, adopting a problem-solving strategy can be maladaptive because the individual may continue to try to correct something that cannot be fixed [38].

Considering that governmental restrictions are the largest source of distress reported by our participants, with visiting restrictions and social isolation being the most important subthemes of the category, it seems hardly surprising that their greatest source of support tended to be support within their relationships or families, as well as contact with their friends and colleagues. This finding corroborates previous research that pointed out that relationships played a relevant role in providing support and maintaining mental balance during the first lockdown in Austria [23,24]. Mariani et al. [25], who explored the effect of coping strategies and perceived social support on depressive and anxious symptomatology during the COVID-19 pandemic in Italy, also found that family support had an exclusive role in mitigating depressive symptoms as it reduced people’s sense of loneliness.

Engaging in recreational activities, which was the second strongest category of support, entailed both indoor and outdoor exercise (10.9%) as well as a range of hobbies (7%). We find it surprising that only 11.1% of our study participants reported finding relief in physical activity since data from Austria showed that exercise could buffer negative effects on mental health [21] and data from Italy showed that those who exercised almost every day during the pandemic were in the best mood, regardless of whether or not they exercised before the pandemic [39]. An explanation could be found in our study design, as we only asked for the most important source of support.

As one would expect, a coping strategy of alcohol and cigarette consumption, as mentioned by 1.5% in our sample (as a subcategory of the avoidance category “distraction”, 10.9%), was found by Budimir et al. to be a small but significant predictor of lower psychological life quality and well-being and higher perceived stress, depression, anxiety, and insomnia during the first lockdown in Austria.

Positive thinking (8.4%) or trust in oneself (5.2%), on the other hand, involves being aware of and thus approaching the situation while attempting not to be overwhelmed by emotions, while conscious relaxation by meditating, breathing exercises, or other self-care activities (8.4%) also involves a component of self-compassion. This means that the individual acknowledges and tries to understand their emotions while at the same time treating themselves kindly [38]. In their study on the effects of different coping strategies on mental health during the first COVID-19 lockdown in Austria, Budimir et al. [22] found that positive thinking was the strongest predictor for psychological well-being, followed by social support, thus indicating that these coping strategies were the most beneficial for mental health. What was less expected, on the other hand, is that it appears that support from faith, as reported by 2.1% in our sample, did not show a negative association in their study between religiosity and anxiety. Instead, it seemed to be a predictor for higher depression and anxiety, which also had a significant predictive value for higher perceived stress and insomnia. The authors explain this result by the fact that individuals who mentioned support from their faith were not able to apply this coping strategy in the usual way during a COVID-19 lockdown as praying with others in religious ceremonies was not possible.

We conclude that the sources of support mentioned by our participants tended to be emotion-focused and associated with a style of emotional approach coping in the sense that they were mostly addressed by actively processing negative emotions. Previous studies have shown some of them to be particularly helpful in reducing stress, such as maintaining social relationships, positive thinking, and sports. Avoidant strategies, on the other hand, as subsumed under the category “distraction”, may lead to more distress instead of less [40].

### 4.3. Limitations

A limitation is that our study participants were required to write their answers in response to a written question instead of being interviewed face to face, which restricted our possibility of deriving more coherent and contextually embedded information from them.

Moreover, all the measurements were self-reported and taken at a single point in time, which may have caused bias, particularly regarding recall and the ability to verbalize. We nevertheless assume that the beginning of the pandemic and thus associated changes in people’s lives should be remembered with certain accuracy. Not only were events unexpected and unique and thus memories thereof not susceptible to inference [41], but it also has been shown that negatively arousing experiences are more likely to be remembered than positive or neutral ones [42].

Furthermore, although we calculated the frequencies of our coded categories to add weight to their meaning and importance, we would like to point out that we did not use standardized scales. While this allowed us to collect authentic answers that we think reflect what our study participants truly found to be the most pressing issues, it also yielded variability between answers in terms of the amount of text, which led to the statements of a minority of study participants being weighted more than once, thus limiting the reliability of our quantification.

Moreover, although two analysts worked on the analysis of the data, it was only in the second step of content analysis that we worked on the same material independently of one another, since we merged the larger number of codes into a smaller number of more abstract categories.

We did not look for gender differences in our data, although these are a likely finding regarding both perceived stressors and support. For future research, we recommend studies with smaller samples composed of more vulnerable members of subgroups as identified by Pieh, Budimir, and Probst [17], using in-depth face-to-face interviews that offer more possibilities of contextualization with regard to causes, context, phenomena, intervening conditions, strategies, and consequences of the COVID-19 experience [43], which also look for gender differences.

A strength of our study is that we used a large sample that has a high degree of representability in relation to the population as a whole, although it should be noted that some subgroups did not achieve the desired quota for a representative sample. In addition, we provide first answers as to what concerns, problem areas, and worries and what means of support are to the fore in a population after almost one year of experiencing COVID-19, a population whose mental health is showing a trend toward deterioration [21].

## 5. Conclusions

Our approach taken here, a qualitative content analysis followed by a quantification of the data, provides empirical evidence primarily regarding the experience of a second “hard” lockdown as the intervention by the public health authorities to combat the spread of COVID-19 at the end of the first year of the COVID-19 pandemic in Austria. A secondary goal was to also look back at problem areas since the beginning of the pandemic in Austria and at worries our participants thought of when anticipating the future.

By employing open-ended questions, we found that social contacts inside and outside the family are of great importance, both in response to the question of the greatest stressors in the present and the past and in response to the question about what helped our participants most. Since the physical distance between people has increased, finding ways to maintain social connectedness is particularly critical [44]. Activating social networks, albeit remotely, is thus not just a key priority but essential in times of isolation [15,45]. Our findings also have implications for future risk communication, as they suggest that a combination of repeated high numbers of infections, ongoing containment measures including social distancing, and the deteriorating mental health of a population that is surrounded by worrisome media reports may yield frustration and dark fantasies about the future [30], such as public health measures being an instrument of oppression or likely to destroy democratic institutions.

We propose more detailed face-to-face qualitative research, in particular, so as to collect the personal experience of more vulnerable groups such as young people, women, and the unemployed.

## Data Availability

Data are available upon reasonable request.

## Supplementary Materials

The following are available online at https://www.mdpi.com/article/10.3390/ijerph18158218/s1, Table S1: The percentages of the sample for each category for the actual sample (N = 1505) and what would be required for representativeness.
