# Supplementary material for: Concerns and Support after One Year of COVID-19 in Austria: A Qualitative Study Using Content Analysis with 1505 Participants
Source: Int J Environ Res Public Health. 2021 Aug 3;18(15):8218. doi: 10.3390/ijerph18158218 (PMC8346103; doi:10.3390/ijerph18158218)
Supplement: Supplementary file 1 [file ijerph-18-08218-s001.zip › ijerph-1236120-supplementary.pdf]

Supplementary Materials

Table S1

Table S1: The percentages of the sample for each category for the actual sample (N=1505) and what would be required for representativeness.

Quota	Final %	Desired %	Difference in Quotas
Overall	100%	100%	0.00%
Male	49.24%	49.50%	-0.26%
Female	50.76%	50.50%	0.26%
Male 18-24	4.19%	5.50%	-1.31%
Female 18-24	5.98%	5.63%	0.36%
Male 25-34	8.64%	9.13%	-0.49%
Female 25-34	9.90%	9.31%	0.59%
Male 35-44	9.50%	8.94%	0.56%
Female 35-44	9.70%	9.13%	0.58%
Male 45-54	11.03%	10.81%	0.22%
Female 45-54	10.63%	11.06%	-0.43%
Male 55-64	9.10%	8.56%	0.54%
Female 55-64	8.97%	8.69%	0.28%
Male 65+	6.78%	6.38%	0.40%
Female 65+	5.58%	6.56%	-0.98%
Burgenland	3.85%	3.63%	0.23%
Lower Austria	20.27%	19.06%	1.20%
Wien	22.99%	23.44%	-0.45%
Carinthia	6.84%	6.50%	0.34%
Steiermark	14.68%	14.19%	0.50%
Oberoesterreich	15.08%	16.69%	-1.60%
Salzburg	5.25%	6.31%	-1.06%
Tirol	7.57%	8.50%	-0.93%
Voralberg	3.46%	4.44%	-0.98%
Low Education	2.66%	2.50%	0.16%
Mid Education	53.75%	50.00%	3.75%
High Education	43.59%	53.06%	-9.47%
